# Performance of a 70-mer oligonucleotide microarray for genotyping of *Campylobacter jejuni*

**DOI:** 10.1186/1471-2180-8-73

**Published:** 2008-05-08

**Authors:** Sandra Rodin, Anders F Andersson, Valtteri Wirta, Lena Eriksson, Marianne Ljungström, Britta Björkholm, Hans Lindmark, Lars Engstrand

**Affiliations:** 1 Swedish Institute for Infectious Disease Control, SE-17182 Solna, Sweden; 2 Limnology/Department of Ecology & Evolution, Uppsala University, Box 573, SE-75123 Uppsala, Sweden; 3 Department of Microbiology, Tumor and Cell Biology, Karolinska Institutet, SE-17177 Stockholm, Sweden; 4 National Food Administration, P.O. Box 622, SE-75126 Uppsala, Sweden

## Abstract

**Background:**

*Campylobacter jejuni *is widespread in the environment and is the major cause of bacterial gastroenteritis in humans. In the present study we use microarray-based comparative genomic hybridizations (CGH), pulsed-field gel electrophoresis (PFGE) and multilocus sequence typing (MLST) to analyze closely related *C. jejuni *isolates from chicken and human infection.

**Results:**

With the exception of one isolate, the microarray data clusters the isolates according to the five groups determined by PFGE. In contrast, MLST defines only three genotypes among the isolates, indicating a lower resolution. All methods show that there is no inherit difference between isolates infecting humans and chicken, suggesting a common underlying population of *C. jejuni*. We further identify regions that frequently differ between isolates, including both previously described and novel regions. Finally, we show that genes that belong to certain functional groups differ between isolates more often than expected by chance.

**Conclusion:**

In this study we demonstrated the utility of 70-mer oligonucleotide microarrays for genotyping of *Campylobacter jejuni *isolates, with resolution outperforming MLST.

## Background

*Campylobacter jejuni *is a major cause of human bacterial gastroenteritis in industrialized countries [1]. Infection commonly results in self-limiting gastroenteritis but sequelae may occur, for instance in the form of the Guillain-Barré syndrome causing peripheral neuropathy [2]. The genus Campylobacter is widespread in the environment and constitutes part of the normal flora of birds, cattle and swine. Although there are gaps in our knowledge of the sources of infection, the handling and consumption of chicken meat are considered important routes of transmission [3,4].

Cases of campylobacteriosis are mainly sporadic but outbreaks do occur, predominantly through contaminated milk and untreated water [5]. Due to the sporadic nature of campylobacter infections, it has proven hard to discern the epidemiological characteristics of the disease. Robust and reproducible typing methods are needed to this end, and a multitude of genotypic methods are now complementing serotyping and other traditional phenotypic methods (for a review, see [6]). Among these, pulsed-field gel electrophoresis (PFGE) possess high discriminatory power and is widely used for studies of strain relatedness [7-11]. However, PFGE requires strict adherence to standardized protocols, and produces data in the form of band patterns of restriction endonuclease digested fragments which are not readily compared between laboratories. Errors or ambiguities in the assignment of bands may also occur [12,13].

A multilocus sequence typing (MLST) scheme assesses genetic differences by nucleotide sequence determination of approximately 500 bases in each of seven loci [14]. The strain discriminatory performance is highly dependent on the screened loci, which are selected to represent slowly evolving genes under stabilizing selection pressure, supposedly unaffected by antigen variation or genomic rearrangements. Each allele is assigned a number based on sequences in the MLST database [15]. Thus, each isolate is described by a seven-digit sequence type (ST), which is further grouped according to lineage into clonal complexes, defined as groups of isolates with identical alleles at ≥4 loci. The MLST scheme has been used in studies of the population structure of clinical and veterinary isolates of *C. jejuni *[10,16-18]. The discriminatory power was comparable to that of multilocus enzyme electrophoresis [10], and amplified fragment length polymorphism [18], but did not reach that of PFGE in a study of epidemiologically related isolates [19].

Comparative genomic hybridizations (CGH) using genome-wide DNA microarrays have proven useful in studies of intraspecies diversity for a number of bacterial species [20-23]. Determination of the full genome sequence of *C. jejuni *strain NCTC 11168 [24] allowed construction of microarrays for studies of the genetic relationship between campylobacter. Using strain NCTC 11168 as reference, several studies have demonstrated a high degree of intraspecies variability concentrated to defined genomic regions, particularly affecting loci coding for lipooligosaccharides, flagellar modification, and DNA restriction-modification systems [25-31]. CGH may also elucidate sources of infection, transmission routes and virulence of bacteria [31,32].

Few studies have exploited the power of CGH to evaluate the accuracy and resolution of present genotyping technologies. In the current study we used a whole-genome microarray to study *C. jejuni *isolates typed with PFGE. We studied closely related pairs of chicken and human isolates, which clustered together in the PFGE analysis, with the aim to dissect the true genetic relationship within and between the pairs. The CGH data in this study were generated using an oligonucleotide array, which was evaluated for its ability to discriminate between present and absent or divergent genes. The results were further compared with MLST results to evaluate the genotyping resolution of the different methods.

## Results

### Multilocus sequence typing

**Figure 1:**
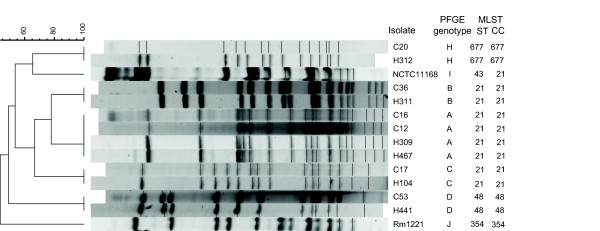
Pulsed-field gel electrophoresis profiles from digestion with *Kpn*I. Isolates denoted "C" originate from chicken, and "H" from human infection. The dendrogram was constructed using the Dice coefficient and the unweighted pair-group method with arithmetric means. Multilocus sequence type (ST) and clonal complex (CC) data of the isolates is also given. The MLST data for RM1221 and NCTC 11168 are from references [36] and [14], respectively.

The twelve isolates representing five distinct PFGE genotypes were analyzed using MLST [14]. Three different STs belonging to three clonal complexes were found among the isolates (Figure 1). Human and chicken isolates of the same PFGE genotype also had the same ST. All isolates within each ST shared the alleles in all seven loci investigated (data not shown).

### Microarray-based genotyping

**Figure 2:**
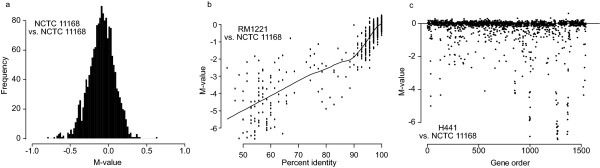
Comparative genomic hybridization data. (A) Histogram of the log_2 _ratio values (M-values) from one of the NCTC 11168 vs. NCTC 11168 (self-self) hybridizations, providing an estimate of the noise level associated with DNA extraction, labeling and hybridization. (B) The effect of probe-target sequence similarity on M-values using data from the RM1221 vs. NCTC 11168 comparison. The line is a locally weighted regression curve generated using the lowess function. (C) M-values vs. probe order according to position in the NCTC 11168 genome showed some regional clustering of divergent or absent genes to genomic hotspots for high variability. Data shown for isolate H441.

On average 1,477 non-redundant probes (92%: range from 1,408 to 1,499) remained after the data preprocessing step and generated a valid log_2 _ratio (M-value). Several of the excluded probes had low signal intensities in both channels and represented probes that failed during array production. To estimate the noise level associated with the experiment and to define M-value thresholds for sequence divergence, we analyzed the M-value distribution of two self-self hybridizations of reference strain DNA prepared at two separate occasions (Figure 2A). As expected for a data set with low technical noise, these hybridizations had narrow M-value distributions (sd = 0.17 and 0.14). Only a few probes had absolute M-values of >0.5 (5 probes (0.3%) for the first hybridization, and 22 (1.3%) for the second hybridization). One single probe (0.06%) in the second hybridization had an absolute M-value of >0.75.

Next, a comparison between the sequenced strains NCTC 11168 and RM1221 allowed for estimation of the effect of sequence divergence on the M-values. We used sequence similarity searches to match each probe with the genome sequence of the RM1221 strain and to calculate the number of matching nucleotides (all probes have a 100% match in the genome of NCTC 11168). There were in total 160 probes (10%) with less than 55 matching bases, putatively measuring highly divergent genes or genomic regions absent in RM1221. Approximately 51% (811/1,601) of the probes had a perfect match in RM1221, while 21% (336/1,601) had a one-base mismatch. A reduction in the number of matching bases displayed a strong association with reduced hybridization signals, and hence a trend towards lower M-values in the comparison between RM1221 and NCTC 11168 (Figure 2B, Table 1). Interestingly, already a one-base divergence resulted in a significant change in the M-values at the global level (shift of mean value from 0.02 to -0.12; p < 10^-16^, one-sided, two-sample t-test).

**Table 1:** Summary of the RM1221 vs. NCTC 11168 comparison and the effect of probe-target similarity on the M-values

			**M-value cut-off**		
			
**Sequence identity**	**Number of probes**	**Mean (SD)**	**<-0.5**	**<-0.75**	**<-1.0**
100	777	0.06 (0.17)	2 (0%)	0 (0%)	0 (0%)
99	315	-0.12 (0.24)	22 (7%)	4 (1%)	2 (1%)
97	128	-0.41 (0.34)	48 (38%)	18 (14%)	6 (5%)
96	60	-0.77 (0.59)	41 (68%)	24 (40%)	19 (32%)
94	40	-1.26 (0.56)	36 (90%)	30 (75%)	27 (68%)
90 – 93	31	-1.53 (0.62)	29 (94%)	27 (87%)	27 (87%)
< = 89	119	-4.02 (1.47)	119 (100%)	119 (100%)	118 (99%)

**Figure 3:**
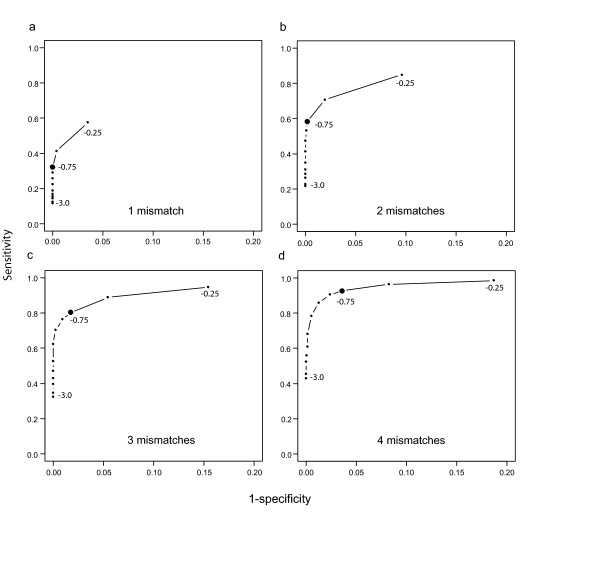
The effect of M-value threshold on sensitivity and specificity for detection of different degrees of probe-target mismatch: (A) ≥ 1 mismatch (B) ≥ 2 mismatches (C) ≥ 3 mismatches and (D) ≥ 4 mismatches. M-values ranging from -3.0 to -0.25 are plotted with an interval of -0.25. An M-value of <-0.75 was chosen for classifying a probe as divergent compared to the sequence of reference strain NCTC 11168.

We further used the RM1221 vs. NCTC 11168 hybridization data to define an M-value threshold for detection of divergent genes. The effect of M-value threshold on sensitivity and specificity for detection of probes with at least one, two, three or four mismatches to the reference strain was plotted (Figure 3). We used this information, in combination with the self-self hybridization data, to define a conservative M-value threshold of less than -0.75 for divergent or absent genes. At this stringency level, none of the probes with a perfect match in the RM1221 genome were erroneously identified as divergent in comparison with NCTC 11168. On the other hand, using this M-value cutoff only 32% of the probes with ≥1 mismatch could be detected as divergent (Figure 3A). The fraction of correctly identified divergent probes increased to 93% when the analysis was restricted to probes with at least four mismatches (94% sequence identity, Figure 3D). Collectively, the analyses indicate that the oligonucleotide platform does not permit reliable genotyping at the single-base level, but can with high confidence be used to identify absent or divergent genes (≥4 mismatches, i.e. sequence identity ≤94%). At this level the probability of false-positives is low and the sensitivity to detect ≥ 4-bp mismatches is high (>93%), providing a reasonable balance between false positives and false negatives.

The isolates included in the study showed large differences in the number of probes (range from 30 to 420) that were sequence divergent compared to the reference strain. In total 29% (439/1,527) and 17% (253/1,527) of the probes were variable in more than two and three of the studied isolates, respectively. These numbers are in line with previous CGH studies [25-31]. The variable probes represented genes distributed over the entire chromosome, and showed some local clustering (Figure 2C). Probes exhibiting M-values of >0.75 were classified as representing genes with higher copy numbers in the test isolate compared to the reference strain NCTC 11168. Genes with higher copy numbers were detected in five of the test isolates (three genes in C12, one in C36, nine in H467, six in C20, and two in H312). In isolates C12 and C20 two consecutive genes with higher copy numbers could be detected (C12: Cj0078c and Cj0079c, C20: Cj0967 and Cj0968, and Cj1419c and Cj1420c). The M-values for all probes are available through the ArrayExpress data repository (accession number E-TABM-460).

### Clustering and correlation of the different typing methods

**Figure 4:**
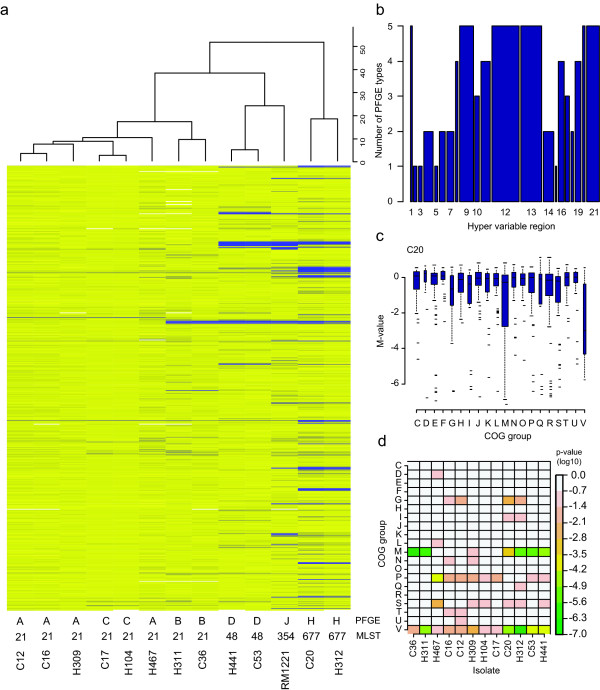
Analysis of variable regions in the *C. jejuni *genome. (A) Hierarchical clustering (Euclidian distance, average linking) based on the entire CGH dataset. The probes are ordered according to their position in the genome, starting with the probe targeting gene Cj0001 at the bottom of the figure. The log_2 _ratio (M-values) of each probe is represented using a color gradient from yellow to blue, denoting M-values ranging from 0 to -7. A negative M-value indicates that the probe shows sequence divergence or absence in the test isolate compared to the NCTC 11168 reference strain. The PFGE genotypes and MLST sequence types are shown below. (B) Presence of variable regions in the analyzed isolates. On the y-axis are the five types of isolates (A, B, C, D and H) included in the study. The height of the bars corresponds to the number of isolate types in which the variable region was identified. The width of each bar is drawn proportional to the number of genes in the corresponding variable region. Regions 1–16 were described by Taboada et al. [27] and regions 17–18 by Parker et al. [29]. Regions 19–21 were identified in this study. (C) Analysis of the divergent genes shows that these represent multiple COG groups, as exemplified for isolate C20. Description of the COG groups is available through the COG database [33]. (D) The representation of COG groups among the variable genes was analyzed using Fisher exact test and the results are summarized using color coding. Groups M and V were significantly overrepresented among the variable genes in multiple isolates.

We next carried out a hierarchical clustering analysis using the microarray data to identify similarities among the isolates. The origin of the isolates (chicken or human) had no effect on the clustering. Instead, the isolates clustered into groups similar to those obtained by PFGE and MLST. Three major clusters were identified (Figure 4A). The first included all isolates with PFGE types A, B and C, belonging to the MLST ST-21 clonal complex. Within this cluster, the two isolates of PFGE type B clustered together, while another cluster was formed by the two type C and three type A isolates. The remaining type A isolate clustered outside this tight cluster. The microarray data indicated that this cluster of isolates is similar to the reference strain NCTC 11168 (Figure 4A). This relatedness is further supported by the MLST data; strain NCTC 11168 belongs to the same clonal complex as the type A, B and C isolates (ST-21 clonal complex), although it is of a different sequence type (ST-43). The second cluster included PFGE type D isolates (MLST clonal complex ST-677) and strain RM1221, and the third PFGE type H isolates (MLST clonal complex ST-48).

### Identification of variable regions

Previous CGH studies of *C. jejuni *have identified 18 genomic regions enriched for genes with diverging sequences [27,29]. We analyzed the presence of variable genes in the five groups of isolates (A, B, C, D and H) defined by PFGE and microarray clustering. A region was confirmed variable if the calculated average M-value of the group was <-0.75 for at least one of the probes in the region. Using this approach, we noted that all of the previously identified variable regions differed in at least one of the five isolate groups, and further that four variable regions (regions 1, 9, 12 and 13) showed divergence in all five groups (Figure 4B). Also region 11 was highly variable, with four isolate groups showing variability in the region. We further mined our data for additional regions that showed variability in multiple isolates. We found three additional regions, spanning genes Cj0137–Cj0145 (region 19), Cj0356c-Cj0360 (region 20) and Cj1047c-Cj1069 (region 21).

### Identification of COG groups enriched for variable regions

We further used the Clusters of Orthologous Groups of proteins (COG) database [33] to analyze the functional group assignments of the variable genes (M-values of <-0.75 for the corresponding probes). In all isolates genes from multiple COG categories were found variable, indicating that sequence divergence is not restricted to genes encoding specific functions (Figure 4C). Furthermore, using Fisher exact test we identified a significant overrepresentation of divergent genes in the COG category M (cell wall/membrane/envelope biogenesis) and V (defense mechanisms) in several of the isolates (Figure 4D). A strong enrichment of the same categories was observed when the analysis was restricted to genes with high sequence divergence (i.e., M-values of <-2.0). However, when the analysis was carried out using genes with moderate sequence divergence (M-values between -2.0 and -0.75), no significant enrichment of COG categories could be observed. These results suggest that the moderate sequence divergence reflects normal interstrain variability unlikely to affect protein function in any substantial way. Furthermore, the probes with moderate sequence divergence seem to be distributed over the entire length of the chromosome, while the probes with high sequence divergence seem to be more tightly clustered (Figure 2C).

## Discussion

In this study we evaluated three different methods for analysis of the genomic content of closely-related *C. jejuni *isolates from chicken and humans. The methods tested were CGH using oligonucleotide microarrays, and genotyping by PFGE and by MLST. We first analyzed six pairs of human and chicken isolates which were clustered based on PFGE of *Kpn*I digests. Cluster analysis based on CGH data yielded an identical grouping, with the exception of one isolate. Thus PFGE, which may appear a relatively crude method, produced a phylogenetic tree which coincided well with the one produced by genomic probing through CGH. The data further suggests that there are no genetic markers distinguishing the human from the chicken isolates included in this study. The isolates were subtyped using a previously described MLST scheme [14]. MLST defined three genotypes among the twelve isolates, compared to five defined by PFGE. All eight isolates with PFGE genotypes A (n = 4), B (2) and C (2) were found to belong to sequence type 21. The ST-21 complex has previously been shown to be abundant among isolates from a wide variety of sources [14,16-19]. The two PFGE genotype D isolates were assigned to ST-48, a sequence type differing from ST-21 in three of the seven loci. Thus, our results suggest that a combined approach using MLST in combination with a second method is necessary to reach a sufficient discriminatory power, at least for resolving epidemiological relationships on a shorter time scale. This conclusion is supported by previous studies [11,34].

Using the microarray data, we have shown that several previously identified regions [27,29] are also divergent in isolates investigated in this study. These include regions that are known to be important modulators of the surface-exposed antigenic proteins (e.g., contain genes encoding flagella proteins). In the present study we identified additional regions that are divergent between the isolates, which suggests that additional genome-wide studies are required to fully characterize the variability of the *C. jejuni *genome. A functional analysis of the variable genes showed that modulation of the surface exposed structures is important for creating variability in the *C. jejuni *isolates, possibly providing means for avoidance of the host's immune system.

As far as we are aware, this is the first study where a genome-wide oligonucleotide array is used for CGH-based genotyping of *C. jejuni*. Previous studies have used microarrays based on polymerase chain reaction-amplified probes for analysis of different *C. jejuni *isolates [25-31]. The main advantage associated with the use of oligonucleotide arrays is the avoidance of extensive cross-hybridization with other regions of the genome and an improved specificity and resolution, allowing detection of smaller differences between the isolates. Also, the design of the probes can be carried out to ensure approximately equal optimal hybridization conditions, avoiding sequence-specific bias in the hybridization signals. However, there are limitations with the oligonucleotide-based CGH platform. The array probes are targeted towards coding regions of the *C. jejuni *genome, which does not allow for detection of divergence in intergenic regions. Although more specific, the oligonucleotide probes do not allow for detection of single-base changes and lack the possibility to detect short deletions and changes in gene synteny. Also, sequence divergence affecting a non-targeted region of a gene will remain undetected using the oligonucleotide probe approach, suggesting that the true differences between the isolates may be even stronger than reported here. As with all microarrays, the analysis is limited to genes represented on the microarray, in this case genes present in strain NCTC 11168. The design could be improved by adding probes representing genes from other sequenced *C. jejuni *genomes.

## Conclusion

In this study we have investigated the variability of closely related *C. jejuni *isolates. The comparative genomics hybridization data did not affect the PFGE-based clustering, with the exeption of one isolate which was removed from the fork containing the remaining isolates of the same PFGE type. Nor did we identify any markers predictive of source (human or chicken). We have further shown that MLST-based genotyping needs to be complemented with other methods to achieve similar resolution as is obtained with the other genotyping approaches. We have also demonstrated that extensive variability between isolates is not restricted to the previously identified regions. Finally, certain functional groups (COG groups M and V) show significant enrichment among the variable genes. Collectively, these results demonstrate the importance of unbiased, genome-wide approaches in analysis of differences between isolates of *C. jejuni*. This will facilitate our future understanding of parameters governing the pathogenic potential of various isolates and allow the design of relevant tools for assessing the genetic diversity and epidemiology of *C. jejuni*.

## Methods

### Campylobacter isolates and extraction of genomic DNA

*Campylobacter spp. *isolates (n = 90) were collected from all reported cases of domestically acquired campylobacteriosis in four Swedish regions within the scope of a study conducted in July through October 2003. During the same time period and in the same geographical areas, fresh poultry products from retail were purchased and analyzed for campylobacter. Isolates from both patients and poultry were species identified by polymerase chain reaction [35] and the *C. jejuni *isolates subtyped using PFGE and the restriction enzyme *Sma*I as earlier described [7]. Isolates sharing PFGE genotype with at least one other isolate were further PFGE-genotyped using the restriction enzyme *Kpn*I. Each unique banding pattern was assigned an identifying letter. For the current study, six pairs of *C. jejuni *isolates with identical *Sma*I and *Kpn*I genotypes were selected to represent each of the *Kpn*I genotypes A (two pairs), B, C, D and H (Figure 1). Each pair consisted of one chicken and one human isolate originating from the same geographical region. The two completely sequenced strains NCTC 11168 [24] and RM1221 [36] were also included.

All isolates were cultured on blood agar plates at 37°C for 48 h in a microaerophilic environment. Genomic DNA for MLST and microarray analyses was extracted using the DNeasy tissue kit (Qiagen, Hilden, Germany).

### Multilocus sequence typing

The seven loci used in the MLST [14] were polymerase chain reaction amplified using primers and conditions according to the *C. jejuni *MLST website . Nucleotide sequences were obtained by sequencing of both strands using the BigDye Terminator v3.1 Cycle Sequencing kit and a 3130xl Genetic Analyzer (Applied Biosystems, Foster City, USA). Sequences were assigned allele numbers, and the sequence type (ST) and lineage (clonal complex) of each isolate was determined by interrogating the MLST database.

### Comparative genomic hybridizations

*Campylobacter jejuni *AROS v1.0 oligonucleotide probe set was purchased from Operon Biotechnologies (Cologne, Germany). The set consisted of 70-mer oligonucleotides representing 1,546 open reading frames (ORFs) from strain NCTC 11168, 51 ORFs from the virulence plasmid pVir from strain 81–176, and 4 ORFs from plasmid pCJ01 from strain 21190. The oligonucleotides were printed in triplicates on CodeLink Activated slides (GE Healthcare, Uppsala, Sweden) and processed according to the manufacturer's instructions. For probe annotation the version 1.3.2 (dated May 16, 2007) of the OMAD database [37] was used. Additional details on the microarray production are available through the ArrayExpress microarray data repository (accession number A-MEXP-925) [38].

Test DNA extracted from the twelve *C. jejuni *isolates was co-hybridized with reference DNA extracted from strain NCTC 11168 in one hybridization per isolate. Additional hybridizations were performed to compare strains NCTC 11168 and RM1221, and to compare two separate target preparations (culturing, DNA extraction, labeling and hybridization) of the NCTC 11168 strain to establish the magnitude of technical noise in the experimental setup. Both sets of additional hybridizations were carried out in a dye-swap manner.

For labeling, 3 μg genomic DNA in 21 μL water was sonicated for 30 s. Fluorescent Cy3 or Cy5 dyes were incorporated in a mixture of 15 μg random octamers, 40 U of Klenow enzyme (Invitrogen, Paisley, UK), 6 nmol of each dATP, dGTP and dCTP, 3 nmol dUTP, and 50 nmol Cy3-/Cy5-dUTP (GE Healthcare, Uppsala, Sweden) in 1 mM Tris pH 8.0, 100 μM EDTA. The mixture was incubated at 37°C for 2 h after which 5 μL 0.5 M EDTA was added. Unincorporated dye was removed using the Microcon-30 columns (Millipore AB, Solna, Sweden) and the dye incorporation efficiency measured using a Nanodrop ND-1000 spectrophotometer (Nanodrop, Rockland, USA). Reference DNA was combined with an equal amount of reciprocally labeled test DNA, dried down using a speed vac, resuspended in 100 μL hybridization buffer (5 × SSC, 50% formamide, 0.1% SDS, 0.1 μg/μL tRNA), and denatured for 2 min at 95°C. The samples were cooled on ice, transferred to the microarrays and hybridized for 16 h at 42°C. The arrays were then washed once for 5 min with 2 × SSC, 0.1% SDS at 42°C, once for 5 min with 0.1 × SSC, 0.1% SDS at room temperature, and four times for one minute with 0.1 × SSC at room temperature. All washing steps were done under agitation. The slides were dried by brief centrifugation at low speed.

The microarrays were scanned using a GenePix 4000B scanner (Molecular Devices, Sunnyvale, USA). Features were identified and fluorescence intensities extracted using the irregular feature-finding approach implemented in GenePix Pro 5.1 (Molecular Devices). Further analysis was carried out in the R environment for statistical computing [39] using the aroma [40], Bioconductor [41] and kth-packages [42]. No subtraction of the local background was carried out, as this was found to slightly increase the variability between replicated features. A feature was considered unreliable and removed if: a) the feature contained less than 55 pixels, or if for both channels b) 10% or more of the pixels were below the signal intensity of the local background plus two standard deviations of the background, c) the signal-to-noise ratio was below 3, d) the signal was saturated, or e) the intensity was below the mean signal of negative controls (probes with random sequence). The design of the probe set is based on the genome sequence of the NCTC 11168 strain, and hence absent or sequence divergent genes in the test isolate (labeled in Cy5) compared to the reference strain (Cy3) show negative log_2 _(Cy5/Cy3) ratio values (M-values). Data normalization was carried out in a block-wise manner assuming equal sums of the two channels using a non-divergent set of probes. These probes were obtained after removal of 20% of the probes with the most negative M-values. After normalization, replicates of each probe were averaged, discarding probes that had only one available measurement. The microarray dataset is available through ArrayExpress (accession number E-TABM-460) [38].

## Authors' contributions

SR carried out the molecular studies, and participated in analysis of the data and drafting of the manuscript. AA designed the microarray experiment and participated in analysis of microarray data. VW analyzed the microarray data and drafted the manuscript. LEr, ML and carried out the molecular experiments. BB participated in the design of the study and coordination of the sample logistics. HL designed and coordinated the sample collection. LEn conceived of the study, and participated in its design and coordination. All authors read and approved the final manuscript.

## References

[B1] Altekruse SF, Stern NJ, Fields PI, Swerdlow DL (1999). Campylobacter jejuni – an emerging foodborne pathogen. Emerg Infect Dis.

[B2] Nachamkin I, Allos BM, Ho T (1998). Campylobacter species and Guillain-Barre syndrome. Clin Microbiol Rev.

[B3] Stern NJ, Fedorka-Cray P, Bailey JS, Cox NA, Craven SE, Hiett KL, Musgrove MT, Ladely S, Cosby D, Mead GC (2001). Distribution of Campylobacter spp. in selected U.S. poultry production and processing operations. J Food Prot.

[B4] Studahl A, Andersson Y (2000). Risk factors for indigenous campylobacter infection: a Swedish case-control study. Epidemiol Infect.

[B5] Pebody RG, Ryan MJ, Wall PG (1997). Outbreaks of campylobacter infection: rare events for a common pathogen. Commun Dis Rep CDR Rev.

[B6] Wassenaar TM, Newell DG (2000). Genotyping of Campylobacter spp. Appl Environ Microbiol.

[B7] Lindmark H, Harbom B, Thebo L, Andersson L, Hedin G, Osterman B, Lindberg T, Andersson Y, Westoo A, Olsson Engvall E (2004). Genetic Characterization and Antibiotic Resistance of Campylobacter jejuni Isolated from Meats, Water, and Humans in Sweden. J Clin Microbiol.

[B8] Fitzgerald C, Helsel LO, Nicholson MA, Olsen SJ, Swerdlow DL, Flahart R, Sexton J, Fields PI (2001). Evaluation of Methods for Subtyping Campylobacter jejuni during an Outbreak Involving a Food Handler. J Clin Microbiol.

[B9] Nielsen EM, Engberg J, Fussing V, Petersen L, Brogren C-H, On SLW (2000). Evaluation of Phenotypic and Genotypic Methods for Subtyping Campylobacter jejuni Isolates from Humans, Poultry, and Cattle. J Clin Microbiol.

[B10] Sails AD, Swaminathan B, Fields PI (2003). Clonal complexes of Campylobacter jejuni identified by multilocus sequence typing correlate with strain associations identified by multilocus enzyme electrophoresis. J Clin Microbiol.

[B11] O'Reilly LC, Inglis TJ, Unicomb L (2006). Australian multicentre comparison of subtyping methods for the investigation of Campylobacter infection. Epidemiol Infect.

[B12] Tenover FC, Arbeit RD, Goering RV, Mickelsen PA, Murray BE, Persing DH, Swaminathan B (1995). Interpreting chromosomal DNA restriction patterns produced by pulsed-field gel electrophoresis: criteria for bacterial strain typing. J Clin Microbiol.

[B13] Singer RS, Sischo WM, Carpenter TE (2004). Exploration of biases that affect the interpretation of restriction fragment patterns produced by pulsed-field gel electrophoresis. J Clin Microbiol.

[B14] Dingle KE, Colles FM, Wareing DR, Ure R, Fox AJ, Bolton FE, Bootsma HJ, Willems RJ, Urwin R, Maiden MC (2001). Multilocus sequence typing system for Campylobacter jejuni. J Clin Microbiol.

[B15] Campylobacter jejuni and Campylobacter coli MLST Home Page. http://pubmlst.org/campylobacter/.

[B16] Dingle KE, Colles FM, Ure R, Wagenaar JA, Duim B, Bolton FJ, Fox AJ, Wareing DR, Maiden MC (2002). Molecular characterization of Campylobacter jejuni clones: a basis for epidemiologic investigation. Emerg Infect Dis.

[B17] Manning G, Dowson CG, Bagnall MC, Ahmed IH, West M, Newell DG (2003). Multilocus sequence typing for comparison of veterinary and human isolates of Campylobacter jejuni. Appl Environ Microbiol.

[B18] Schouls LM, Reulen S, Duim B, Wagenaar JA, Willems RJL, Dingle KE, Colles FM, Van Embden JDA (2003). Comparative Genotyping of Campylobacter jejuni by Amplified Fragment Length Polymorphism, Multilocus Sequence Typing, and Short Repeat Sequencing: Strain Diversity, Host Range, and Recombination. J Clin Microbiol.

[B19] Sails AD, Swaminathan B, Fields PI (2003). Utility of multilocus sequence typing as an epidemiological tool for investigation of outbreaks of gastroenteritis caused by Campylobacter jejuni. J Clin Microbiol.

[B20] Bjorkholm B, Lundin A, Sillen A, Guillemin K, Salama N, Rubio C, Gordon JI, Falk P, Engstrand L (2001). Comparison of genetic divergence and fitness between two subclones of Helicobacter pylori. Infect Immun.

[B21] Behr MA, Wilson MA, Gill WP, Salamon H, Schoolnik GK, Rane S, Small PM (1999). Comparative genomics of BCG vaccines by whole-genome DNA microarray. Science.

[B22] Anjum MF, Lucchini S, Thompson A, Hinton JC, Woodward MJ (2003). Comparative genomic indexing reveals the phylogenomics of Escherichia coli pathogens. Infect Immun.

[B23] Edwards RA, Olsen GJ, Maloy SR (2002). Comparative genomics of closely related salmonellae. Trends Microbiol.

[B24] Parkhill J, Wren BW, Mungall K, Ketley JM, Churcher C, Basham D, Chillingworth T, Davies RM, Feltwell T, Holroyd S (2000). The genome sequence of the food-borne pathogen Campylobacter jejuni reveals hypervariable sequences. Nature.

[B25] Leonard EE, Takata T, Blaser MJ, Falkow S, Tompkins LS, Gaynor EC (2003). Use of an open-reading frame-specific Campylobacter jejuni DNA microarray as a new genotyping tool for studying epidemiologically related isolates. J Infect Dis.

[B26] Dorrell N, Mangan JA, Laing KG, Hinds J, Linton D, Al-Ghusein H, Barrell BG, Parkhill J, Stoker NG, Karlyshev AV (2001). Whole Genome Comparison of Campylobacter jejuni Human Isolates Using a Low-Cost Microarray Reveals Extensive Genetic Diversity. Genome Res.

[B27] Taboada EN, Acedillo RR, Carrillo CD, Findlay WA, Medeiros DT, Mykytczuk OL, Roberts MJ, Valencia CA, Farber JM, Nash JHE (2004). Large-Scale Comparative Genomics Meta-Analysis of Campylobacter jejuni Isolates Reveals Low Level of Genome Plasticity. J Clin Microbiol.

[B28] Pearson BM, Pin C, Wright J, I'Anson K, Humphrey T, Wells JM (2003). Comparative genome analysis of Campylobacter jejuni using whole genome DNA microarrays. FEBS Letters.

[B29] Parker CT, Quinones B, Miller WG, Horn ST, Mandrell RE (2006). Comparative Genomic Analysis of Campylobacter jejuni Strains Reveals Diversity Due to Genomic Elements Similar to Those Present in C. jejuni Strain RM1221. J Clin Microbiol.

[B30] Poly F, Threadgill D, Stintzi A (2004). Identification of Campylobacter jejuni ATCC 43431-Specific Genes by Whole Microbial Genome Comparisons. J Bacteriol.

[B31] Champion OL, Gaunt MW, Gundogdu O, Elmi A, Witney AA, Hinds J, Dorrell N, Wren BW (2005). Comparative phylogenomics of the food-borne pathogen Campylobacter jejuni reveals genetic markers predictive of infection source. Proc Natl Acad Sci USA.

[B32] On SL, Dorrell N, Petersen L, Bang DD, Morris S, Forsythe SJ, Wren BW (2006). Numerical analysis of DNA microarray data of Campylobacter jejuni strains correlated with survival, cytolethal distending toxin and haemolysin analyses. Int J Med Microbiol.

[B33] Clusters of Orthologous Groups of proteins. http://www.ncbi.nlm.nih.gov/COG.

[B34] Price EP, Huygens F, Giffard PM (2006). Fingerprinting of Campylobacter jejuni by using resolution-optimized binary gene targets derived from comparative genome hybridization studies. Appl Environ Microbiol.

[B35] Linton D, Lawson AJ, Owen RJ, Stanley J (1997). PCR detection, identification to species level, and fingerprinting of Campylobacter jejuni and Campylobacter coli direct from diarrheic samples. J Clin Microbiol.

[B36] Fouts DE, Mongodin EF, Mandrell RE, Miller WG, Rasko DA, Ravel J, Brinkac LM, DeBoy RT, Parker CT, Daugherty SC (2005). Major structural differences and novel potential virulence mechanisms from the genomes of multiple campylobacter species. PLoS Biol.

[B37] Operon OMAD database. http://www.operon.com.

[B38] ArrayExpress microarray data repository. http://www.ebi.ac.uk/arrayexpress.

[B39] The R project for statistical computing. http://www.r-project.org.

[B40] Bengtsson H aroma – An R Object-oriented Microarray Analysis environment. http://www.maths.lth.se/publications/.

[B41] Gentleman RC, Carey VJ, Bates DM, Bolstad B, Dettling M, Dudoit S, Ellis B, Gautier L, Ge Y, Gentry J (2004). Bioconductor: open software development for computational biology and bioinformatics. Genome Biol.

[B42] Wirta V, Lindberg J, Gry Björklund M, Klevebring D (2004). The kth-package for microarray data analysis. http://www.biotech.kth.se/molbio/microarray.

